# Low Seroprevalence among Undetected COVID-19 Cases, Faroe Islands, November 2020

**DOI:** 10.3201/eid2801.210917

**Published:** 2022-01

**Authors:** Maria Skaalum Petersen, Marin Strøm, Jógvan Páll Fjallsbak, Jóhanna Ljósá Hansen, Sólrun Larsen, Eina H. Eliasen, Malan Johansen, Anna Sofía Veyhe, Marnar Fríðheim Kristiansen, Pál Weihe

**Affiliations:** The Faroese Hospital System, Tórshavn, Faroe Islands (M.S. Petersen, E.H. Eliasen, M. Johansen, A.S. Veyhe, P. Weihe);; University of the Faroe Islands, Tórshavn (M.S. Petersen, M. Strøm, E.H. Eliasen, M. Johansen, A.S. Veyhe, M.F. Kristiansen, P. Weihe);; Faroese Food and Veterinary Authority, Tórshavn (J.P. Fjallsbak, J.L. Hansen, S. Larsen);; National Hospital of the Faroe Islands, Tórshavn (M.F. Kristiansen)

**Keywords:** COVID-19, respiratory infections, severe acute respiratory syndrome coronavirus 2, SARS-CoV-2, SARS, coronavirus disease, zoonoses, viruses, coronavirus, seroprevalence, Faroe Islands

## Abstract

We conducted a second nationwide severe acute respiratory syndrome coronavirus 2 seroprevalence study in the Faroe Islands during November 2020. We found crude seroprevalence was 0.3% and prevalence was 0.4% after adjusting for test sensitivity and specificity. This low seroprevalence supports the prevention strategies used in the Faroe Islands.

Early in the coronavirus disease (COVID-19) pandemic, the World Health Organization recommended close surveillance and abundant testing at the regional level (*1*). In the Faroe Islands, extensive testing capacity, easily accessible testing, and intensive contact tracing helped eliminate COVID-19 after the first (*2*) and second epidemic waves and further contained outbreaks later in 2020 (*3*). A population-based seroprevalence study of 1,075 persons in the Faroe Islands during May 2020 reported few undetected cases (*4*). However, prevalence studies from Spain, Greece, and Denmark measured severe acute respiratory syndrome coronavirus 2 (SARS-CoV-2) antibody seroprevalences of 0.36%–34.6% (*5*–*7*).

During the first COVID-19 wave in the Faroe Islands, societal lockdown and border closings helped contain the contagion. After the first wave, rather than reinstating lockdown, the country implemented testing, tracing, and quarantine, combined with entry restrictions for travelers, including a negative SARS-CoV-2 test upon entry and recommended self-quarantine until retesting 6 days after arrival (*3*). Despite society returning to near prepandemic normal, subsequent outbreaks in the Faroe Islands were contained efficiently. However, the reopening strategy might have led to undetected cases. We conducted a seroprevalence survey to estimate the number of undetected COVID-19 cases in the Faroe Islands.

We randomly selected 1,500 persons from the Faroese Population Registry (https://www.us.fo/Default.aspx?ID=13792). After excluding 2 newborns, we invited 1,498 persons by letter to provide blood samples at 1 of 6 study sites around the islands during November 21–30, 2020. We offered home visits to those unable to attend. Nonresponders received a follow-up phone call. All participants provided oral and written informed consent. The study was approved by the Faroese Ethical Committee and Data Protection Agency and is methodologically aligned with the World Health Organization generic protocol for population-based seroepidemiologic COVID-19 studies (*1*).

We conducted total antibody analyses by using the SARS-CoV-2 Ab ELISA Kit (Beijing Wantai Biologic Pharmacy Enterprise, https://www.ystwt.cn), which has a sensitivity of 94.4% (95% CI 90.9%–96.8%) and specificity of 100% (95% CI 98.8%–100.0%). We estimated 95% CI for crude seroprevalence by using exact binomial models and used bootstrap methods to adjust seroprevalence for test performance (*8*).

In all, 960 (64.1%) persons participated in the serosurvey (Figure); mean age was 48 years (SD 21.0, range 1–98 years), 52.2% were female, and 47.8% were male (Table). We excluded 12 persons with a previous positive reverse transcription PCR (RT-PCR) result from the seroprevalence study but included them in the total number of cases.

**Figure:**
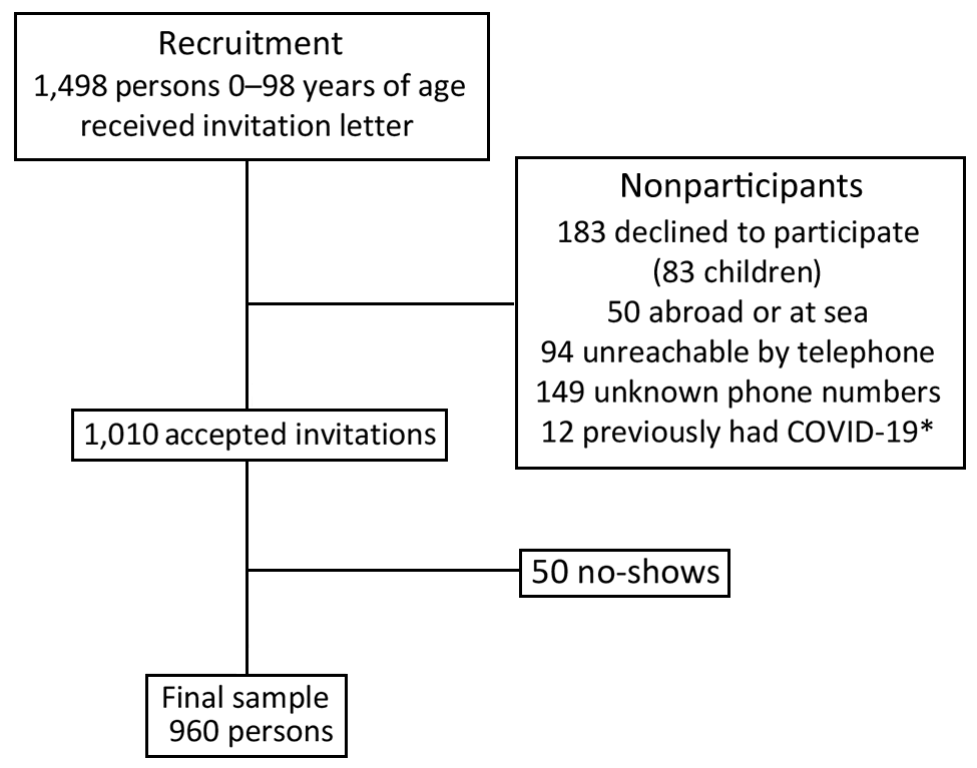
Flowchart of participant recruitment for severe acute respiratory syndrome coronavirus 2 seroprevalence study, Faroe Islands, November 2020. *Persons previously diagnosed with COVID-19 were excluded from serosurvey but included in the total number of cases. COVID-19, coronavirus disease.

**Table:** Characteristics of study participants compared with entire population and crude prevalence for severe acute respiratory syndrome coronavirus 2–specific antibodies, Faroe Islands, November 2020

Characteristics	No. (%) sampled	Total population (%)	p value	No. positive	Crude seroprevalence, % (95% CI)*
Total	960 (100)	52,854 (100)		3	0.3 (0.06–0.9)
Sex					
M	459 (47.8)	27,380 (51.8)	0.014	1	0.2 (0.01–1.2)
F	501 (52.2)	25,474 (48.2)	0.014	2	0.4 (0.05–1.4)
Age, y					
0–9	33 (3.4)	7,259 (13.7)	<0.0001	1	3.0 (0.08–15.8)
10–19	76 (7.9)	7,357 (13.9)	<0.0001	0	0
20–29	88 (9.2)	5,983 (11.3)	0.0414	0	0
30–39	134 (14.0)	6,534 (12.4)	0.1364	0	0
40–49	135 (14.1)	6,554 (12.4)	0.1136	0	0
50–59	191 (19.9)	6,780 (12.8)	<0.0001	0	0
60–69	157 (16.4)	5,685 (10.8)	<0.0001	2	1.3 (0.2–4.5)
70–79	103 (10.7)	4,337 (8.2)	0.0053	0	0
80–89	36 (3.8)	1,875 (3.5)	0.6165	0	0
>90	7 (0.7)	490 (0.9)	0.5147	0	0
Geographic area					
Streymoy	473 (49.3)	25,288 (47.8)	0.3565	2	0.4 (0.05–1.5)
Eysturoy	216 (22.5)	11,966 (22.6)	0.9415	0	0
Norðoyggjar	114 (11.9)	6,278 (11.9)	1	0	0
Vágar	49 (5.1)	3,361 (6.4)	0.1023	0	0
Sandoy og Suðuroy	108 (11.2)	5,961 (11.3)	0.9227	1	0.9 (0.02–5.1)

*Exact 95% CI calculated by binomial regression.

The study sample was geographically representative of the population but had minor differences in sex and age distribution. More men and younger persons comprised nonparticipants than participants: 41.3 (SD +23.4) years of age for nonparticipants versus 48.1 (SD +20.5) years of age for participants (p<0.0001). Persons 0–29 years of age were underrepresented and persons 50–79 years of age overrepresented. 

Among participants, 3 tested positive for SARS-CoV-2–specific antibodies, resulting in a crude seroprevalence of 0.3% (exact binomial 95% CI 0.06%–0.9%). After adjusting for test sensitivity and specificity, we estimated a seroprevalence of 0.4% (bootstrap 95% CI 0.1%–1.0%). Including cases previously confirmed by RT-PCR, seroprevalence in the sample was 1.5%.

We found only a few undetected cases, underpinning the effectiveness of the prevention strategies in the Faroe Islands. Among the 3 seropositive cases, 1 was a child who had experienced symptoms at the beginning of the epidemic. Subsequent serologic analyses revealed that both parents and the child’s siblings were seropositive. The other 2 seropositive cases were in adults who did not recall any symptoms.

Our study’s strengths include the sample size, ≈2% of the country’s population, and the high participation rate of 64%, which increases to 77% when we exclude 243 persons who were not reachable. A study of 82 seroprevalence estimates from 51 different locations and >500 participants noted infection rates ranging from 0.02% to 53.4% by September 9, 2020, but reported large variations in sampling, clustering, and adjustment for test performance (*9*). A serosurvey of 4,000 persons in Switzerland during November–December 2020 reported regional seroprevalence of 21.2% after the second pandemic peak (*10*), >10 times higher than that observed in the Faroe Islands. The differences in seroprevalence might reflect differences in COVID-19 management strategies and geography because, unlike Switzerland, the Faroe Islands do not share borders with other countries. Furthermore, participation rates in the study from Switzerland varied substantially across age groups, from 17% for persons 0–18 years of age to 69% for persons >65 years of age.

In May 2020, we estimated 0.6% seroprevalence in the Faroe Islands (*4*), resulting in slightly higher number of cases than official confirmed cases. Applying the 1.5% seroprevalence we found in this study to the overall population corresponds to 793 cases, whereas 663 RT-PCR–confirmed cases were officially reported (https://www.corona.fo). Nonetheless, our results show that prevention strategies effectively managed the COVID-19 epidemic in the Faroe Islands and that the country effectively reacted to timely information of the contagion.
